# A Survey of Patient-Relevant Outcomes in Pediatric Craniopharyngioma: Focus on Hypothalamic Obesity

**DOI:** 10.3389/fendo.2022.876770

**Published:** 2022-05-09

**Authors:** Meghan Craven, Julia H. Crowley, Lucas Chiang, Cassie Kline, Fatema Malbari, Matthew C. Hocking, Shana E. McCormack

**Affiliations:** ^1^Division of Endocrinology and Diabetes, Department of Pediatrics, Children’s Hospital of Philadelphia, Philadelphia, PA, United States; ^2^Division of Oncology, Department of Pediatrics, Children’s Hospital of Philadelphia, Philadelphia, PA, United States; ^3^Division of Neurology and Developmental Neurosciences, Department of Pediatrics, Texas Children’s Hospital, Houston, TX, United States; ^4^Department of Child and Adolescent Psychiatry and Behavioral Sciences, Children’s Hospital of Philadelphia, Philadelphia, PA, United States

**Keywords:** hypothalamic obesity, pediatric craniopharyngioma, social function, brain tumor, hypopituitarism

## Abstract

**Context:**

Individuals treated for pediatric craniopharyngioma, a rare, grade 1 brain tumor, frequently develop hypothalamic obesity, a complication often recalcitrant to intervention. Although hypothalamic obesity is known to adversely impact quality of life, less is known about how caregivers and patients experience this condition.

**Objective:**

Our goal was to examine the approaches that families take towards weight management and the impact on social function in individuals with craniopharyngioma and obesity. Individuals with craniopharyngioma without obesity were included as a comparison.

**Subjects and Methods:**

Adult caregivers of children <18y with craniopharyngioma completed a web-based survey posted by a patient advocacy organization between February and July 2020. Questions related to the child’s diagnosis, medications, lifestyle modifications, and social function along with research priorities. Descriptive statistics were generated. Linear regression was used to assess the independent effects of obesity and other covariates on social function.

**Results:**

Of 106 respondents, 60 (57%) reported their child had obesity at the time of survey completion. In contrast, only 6 (5.7%) had obesity prior to craniopharyngioma diagnosis. A majority (92%) of those with obesity had tried limiting calories or carbohydrates; 31% and 69% found these helpful, respectively. Thirty-eight percent had tried weight loss medications (stimulants, metformin, GLP1R-agonists, and topiramate) and 48% found at least one helpful. Both stimulant and anti-depressant use were reported more frequently with obesity. An index (T-score) reflecting social function was lower in the cohort than a population reference, 41 (SD 11) vs. 50 (SD 10), p<0.001. In a linear model, both older age and obesity were independently associated with greater social impairment. Ninety-four percent of respondents caring for a child with obesity (and 79% of all respondents) identified “improving treatments and prevention for hypothalamic obesity” as a key research priority.

**Conclusions:**

Only a minority of individuals with hypothalamic obesity had trialed medication, even though many reported that lifestyle modification was inadequate. Furthermore, social function was significantly impaired overall in survivors compared to a reference cohort, and even more so in individuals with obesity. These findings highlight the opportunity to improve social functioning as an additional potential benefit of improved treatments for hypothalamic obesity.

## Introduction

Craniopharyngioma is a rare, WHO grade 1 hypothalamic/pituitary brain tumor with an incidence of 0.5-2.5 per million (1, 2). Children make up approximately 30-50% of these cases and survivors of pediatric craniopharyngioma frequently go on to develop sequelae that adversely impact quality of life, including hypothalamic obesity, pituitary hormone deficiencies, vision impairment, and cognitive deficits (3). Together, these comorbidities can lead to poor social connectedness, reduced independence, difficulty forming relationships, and increased future risk of depression (4, 5).

Prior studies have implicated hypothalamic obesity as not only contributing to impaired quality of life, but also impacting risk for lifelong morbidity and mortality from cardiometabolic disease in survivors of childhood craniopharyngioma (6, 7). Survivors with obesity report lower physical abilities, worse perceptions of body image, and fewer positive social interactions than individuals without obesity (6). In addition to adversely affecting overall quality of life, these feelings might also contribute to individuals being less physically active thereby compounding the adverse metabolic, mental health, and social effects of their condition (8). Moreover, while not universal, hyperphagia is reported by some individuals in the setting of hypothalamic obesity, and poses another therapeutic challenge for affected individuals and families that may also affect social function, for example by leading to avoidance of gatherings where food is widely available (9).

Hypothalamic obesity is often refractory to conventional obesity treatments, although a multi-pronged approach may yield modest benefits as suggested by the available evidence base (10, 11). The International Registry of Hypothalamic Obesity Disorders reported in 2018 on the experiences of individuals with hypothalamic obesity (N=87) due to a variety of conditions, including 86% with brain tumors and 41% under age 20 years, in managing their condition (12). The study found ninety percent received treatment including nutritional counseling (82%), pharmacotherapy (59%), and bariatric surgery (8%), of which surgery was most effective (12). We sought to build on these important observations by focusing specifically on identifying the continued unmet needs in pediatric survivors of childhood craniopharyngioma as reported by their caregivers.

Specifically, our goal in this study was to examine the approaches that families take for weight management, taking into consideration patient-level factors including treatment history, pituitary hormonal deficiencies, and non-hormonal medications. We included survivors of craniopharyngioma without obesity as a comparison and incorporated assessments of social function to capture the associations between weight management efforts and other dimensions of well-being that are relevant to patients. Finally, to examine the extent to which the survey cohort reflects all individuals with craniopharyngioma, we examined the demographic and clinical characteristics of a group of children with craniopharyngioma followed at our institution.

## Methods

### Survey

We invited adult caregivers (≥ 18 years old) of children and adolescents who had been diagnosed with a brain tumor to complete survey questions related to medical history (including treatments trialed for managing excess weight gain), medication use, psychosocial function (peer relationships), and research priorities. The surveys were made available online on social media to community members of the Raymond A. Wood Foundation (a patient advocacy organization) *via* a posted electronic link. Survey data were collected and managed using REDCap (Research Electronic Data Capture), a secure, web-based software platform hosted at CHOP (13, 14). Data were collected between February 12, 2020 and July 17, 2020. For this analysis, we focused on responses from caregivers of children who i) had a history of craniopharyngioma and ii) were under 18 years of age at the time of survey completion.

The majority of demographic responses were chosen from set choices *via* a radio button, drop-down list, or a set of checkboxes when multiple answer choices may apply, including self-reported medical conditions, obesity status, and treatment. All drop-down questions included an “other” option with space to free text answers. (See **Supplementary Material** for full survey.)

The Children’s Hospital of Philadelphia (CHOP) IRB reviewed the study and determined that the study met exempt criteria (IRB 21-018407). No protected health information was collected.

### Medications

The medication and supplement survey asked for caregiver participants to provide free text with each medication/supplement name, indication, and dose with a drop-down menu for dosing units, frequency, and route, as well as a yes/no radio button to indicate if medication was as needed (PRN). The medication dataset was organized first by recoding each free-text medication with its generic name, and then assigning therapeutic drug classifications and indication(s) based on respondent-provided free-text information. Respondent-entered supplements included vitamins and minerals such as vitamin D3, iron, magnesium, potassium, and zinc, and other non-prescription health products including probiotics, supplemental nutrition, and omega-3 fatty acids. To look for differences in prescribing patterns medication and supplement usage, responses were stratified based on both self-reported current obesity status and age (with younger individuals classified as <12 years old and older individuals classified as ≥ 12 years old).

### Peer Relationships

The National Institutes of Health Patient-Reported Outcome Measurement Information System (PROMIS) parent-proxy measure of pediatric peer relationships v.2.0 (15, 16) was used to assess social function. Responses for the PROMIS assessment were compiled for children ages 5-17 years old, the ages for which this questionnaire was designed. The seven-question survey also administered in REDCap utilizing a 5-point Likert scale from “never” to “almost always”. Responses were scored and converted to a standardized T-score with a mean of 50 and standard deviation of 10, where a higher score indicates better peer relationships (https://www.assessmentcenter.net/ac_scoringservice).

### Research Priorities

Respondents were asked to indicate which topic(s) deserved highest priority for future research; respondents could indicate more than one response. There were seven choices plus a space to provide free text with additional responses. Options included: “How can my child’s tumor be more effectively treated and/or how can recurrence be prevented?”, “How can my child’s psychosocial function be improved?”, “How can my child’s fatigue/energy level be improved?”, “How can my child’s pituitary hormonal replacement be improved?”, “How can hypothalamic obesity be prevented or treated?”, “How can my child’s learning deficits be addressed?”, and “How can our family’s function be improved?”

### Statistical Analyses

Summary statistics were generated, including means and standard deviations (SDs) or medians and inter-quartile ranges (IQRs), as appropriate given variable distributions. Proportion of individuals using specific hormonal replacement or other medications were compared between sub-groups of interest (e.g., with vs. without obesity) using Fisher’s exact test. McNemar’s test was used to test for differences between the proportion of individuals who reported being diagnosed with a given hormonal deficiency and the proportion of the same individuals reported receiving hormonal replacement therapy. Sample size was one of convenience. Statistical significance was taken to be a two-sided p-value of <0.05. All statistical analyses were performed in Microsoft Excel and R Studio version 1.4.1103.

### Children’s Hospital of Philadelphia (CHOP) Electronic Medical Record (EMR) Cohort

In order to evaluate the extent to which our survey respondents reflected all individuals with craniopharyngioma, we extracted clinical characteristics from the electronic medical records of all children with craniopharyngioma diagnosed between 2008 and 2020 and receiving care at CHOP (IRB exemption #18407).

## Results

Of the 166 total survey respondents, 137 were caregivers of individuals who had previously been diagnosed with craniopharyngioma and 106 of 137 were caregivers of individuals under 18 years of age at the time of survey completion (**Table 1**). Within the cohort, the average age at craniopharyngioma diagnosis was 7.2y ± 3.2 (SD) and average age at time of survey collection was 11.2y ± 4.1 (SD). More than half, 61% (65/106) were male. With respect to tumor treatment, 93% (99/106) had undergone surgical resection with the majority, 67% (71/106) undergoing open tumor resection and a smaller portion, 36% (38/106) undergoing endoscopic resection (**Table 1**). A smaller proportion were treated with radiation (12%, 13/106) and proton beam therapy (33%, 35/106). Most children (57%, 60/106) had obesity at the time of survey completion while only 5.7% (6/106) had obesity prior to craniopharyngioma diagnosis. Of the children with obesity, there was a higher proportion treated with radiation or proton therapy (58%, 35/60 vs. 28%, 13/46; p=0.003) and a lower proportion treated with endoscopic surgery (22%, 13/60 vs. 54%, 25/46; p<0.001) when compared to children without obesity.

**Table 1:** Patient demographics and clinical characteristics.

Characteristic	Total Patients (%)
**Age at Diagnosis**	
<5 years old	23 (22)
5-11 years old	70 (66)
12-17 years old	11 (10)
Not Reported	2 (2)
**Current Age**	
<5 years old	8 (8)
5-11 years old	49 (46)
12-17 years old	49(46)
**Sex**	
Male	65 (61)
Female	41 (39)
**Obesity Prior to Craniopharyngioma Diagnosis**	
Present	6 (6)
Absent	100 (94)
**Current Obesity Status**	
Present	60 (57)
Absent	46 (43)
**Treatment**	
Endoscopic Surgery	38 (36)
Open Surgery	71 (67)
Cyst Drainage	25 (24)
Radiation	13 (12)
Proton Beam	35 (33)
Chemotherapy	3 (3)

Caregiver reported demographic and clinical characteristics about their child with a history of craniopharyngioma.

All 106 completed the questions on demographics and medical history, 93 completed the peer relationship questionnaire, 69 completed the detailed medication list, and 78 completed the survey on research priorities. The demographics of those who completed the detailed medication and supplement survey group were similar to the study population as a whole. In **Supplementary Table 1**, we show the demographics and clinical characteristics of all survey respondents, as well as the subsets of survey respondents who elected to complete each survey component. For comparison, we also show the demographic characteristics of children with pediatric craniopharyngioma followed at CHOP.

### Pituitary Hormone Deficiencies

Rates of specific caregiver-reported pituitary hormone deficiencies were similar in children with or without obesity (**Table 2**) and were consistent with rates inferred from medication details in the subset of individuals (n=69) who completed both surveys for cortisol, thyroid, and vasopressin deficiency. Of these, 5% (3/56) were receiving combined LT4/T3 for thyroid replacement. Growth hormone deficiency was reported in 83% (57/69), and of those 39% (27/69) reported currently taking growth hormone (p<0.01). In addition, hypogonadotropic hypogonadism was reported in 73% (16/22) and of those, 45% (10/22) reported currently taking sex steroid replacement in the cohort restricted to girls 12 years and older and boys 14 years and older. Three children (3%, 3/106) had no reported pituitary hormone deficiencies.

**Table 2:** Pituitary hormone deficiencies.

Caregiver-reported Hormone Deficiencies (medical history)			
Hormone deficiency	Individuals with obesity (n=60) n (%)	Individuals without obesity (n=46) n (%)	All Individuals(N=106) n (%)
Thyroid	52 (87)	41 (89)	93 (88)
Adrenal	51 (85)	36 (78)	87 (82)
Growth	49 (82)	39 (85)	88 (83)
Gonadal Steroids*	18 (72)	13 (81)	31 (76)
Vasopressin	47 (78)	37 (80)	84 (79)
Pan-hypopituitarism*	34 (57)	26 (57)	60 (57)
**Caregiver-reported Hormone Replacement (medication list)**			
**Hormone replacement**	**Individuals with obesity (n=41)** **n (%)**	**Individuals without obesity (n=28)** **n (%)**	**All** **Individuals** **(N=69)** **n (%)**
Thyroid	35 (85)	21 (75)	56 (81)
Adrenal	34 (83)	17 (61)	51 (74)
Growth	15 (37)	12 (43)	27 (39)
Gonadal Steroids*	6 (40)	4 (57)	10 (45)
Vasopressin	30 (73)	19 (68)	49 (71)
Pan-hypopituitarism*	12 (29)	6 (21)	18 (26)

The prevalence of pituitary hormone deficiencies stratified by current obesity status based on self-reported medical history (top panel) and self-reported medication use (bottom panel). Of note, N=106 caregivers completed the medical history questions, while only a subset of these (N=69 of 106) elected to complete the detailed medication list. No statistically significant differences were detected between individuals with versus without obesity in the prevalence of pituitary hormone deficiencies.

*Evaluated for hypogonadotropic hypogonadism if age ≥12 years for females and ≥14 years for males.

### Other Medications

Children were reported to be taking a median of 4 different medications, including pituitary hormone replacement (IQR 2-6, range 1-12). Medications relevant to the other comorbidities that may be present in individuals with craniopharyngioma, including anti-depressants, stimulants, supplements, and metabolic modifying agents can be found in **Table 3**. All individuals taking anti-depressants were also reported to have obesity. Stimulant use was nominally though not statistically significantly higher in individuals with obesity versus those without (22%, 9/41 vs. 7%, 2/28; p=0.18) and was significantly higher in older children (30%, 8/27 in older individuals vs. 7%, 3/42 in younger individuals, p=0.02). With respect to other non-stimulant medications that could impact weight, individuals were taking metformin (n=6), topiramate (n=5), naltrexone (n=3), and oxytocin (n=1). None were currently taking a GLP-1 receptor agonist, though some had trialed these in the past.

**Table 3:** Non-hormonal caregiver-reported medications of interest.

Medication	Individuals with obesity (n=41) n (%)		Individuals without obesity (n=28) n (%)		All (N=69) n (%)	
Metformin	5	(12)	1	(3.6)	6	(8.7)
<12y	1	(4.3)	0	(0)	1	(2.4)
≥12y	4	(22)	1	(11)	5	(19)
Stimulants	9	(22)	2	(7.1)	11	(16)
<12y	3	(13)	0	(0)	3	(7.1)
≥12y	6	(33)	2	(22)	8	(30)
Anti-depressants	5	(12)	0	(0)	5	(7.2)
<12y	1	(4.3)	0	(0)	1	(2.4)
≥12y	4	(22)	0	(0)	4	(15)
Supplements	14	(34)	9	(32)	23	(33)
<12y	6	(26)	3	(16)	9	(21)
≥12y	8	(44)	6	(67)	14	(52)

Non-hormonal medications summarized from respondents who provided a detailed medication list (N=69), stratified by current obesity status and age [under 12 years old [n=42] compared to 12 years and older (n=27)].

### Efficacy of Weight Management Strategies

Caregiver-reported response to non-pharmacologic and pharmacologic approaches to risk for excess weight gain are shown in **Table 4**. Most caregivers (79%, 84/106) of children with and without obesity had reported limiting intake of overall calories and/or carbohydrates. In general, limiting carbohydrate intake was the lifestyle modification strategy with the most positive response. Of children with obesity, 38% (23/60) had undergone a trial of medication for weight management. All children who trialed a medication has also trialed at least one lifestyle modification, a dietary change plus or minus exercise. Of these, 48% (11/23) found one or more medication helpful. In addition, 24% (11/46) of children without obesity had also undergone a trial of a medication for weight management. There was variability (and few respondents) in the extent to which respondents found medications helpful. As might be expected, caregivers of individuals without obesity reported nominally overall more positive response to interventions than caregivers of those with obesity, though these did not reach statistical significance (72%, 21/29 versus 60%, 33/55; p=0.34 for any dietary modification and 82%, n = 9/11 versus 48%, n=11/23; p=0.08 for any medication).

**Table 4:** Management of risk for excess weight gain.

Intervention	Individuals with obesity (n=60)		Individuals without obesity (n=46)	
	Trialed n (%)	Helpful n (% of trialed)	Trialed n (%)	Helpful n (% of trialed)
**Dietary Treatment (any)**	**55 (92)**	**33 (60)**	**29 (63)**	**21 (72)**
Limiting carbohydrates	45 (75)	31 (69)	25 (54)	17 (68)
Limiting calories	42 (70)	13 (31)	23 (50)	13 (57)
Diet (other)	7 (12)	1 (14)	6 (13)	2 (33)
**Exercise (any)**	**44 (73)**	**23 (52)**	**24 (52)**	**16 (67)**
Independent	41 (68)	21 (51)	22 (48)	13 (59)
Supervised	22 (37)	10 (45)	9 (20)	7 (78)
**Medications (any)**	**23 (38)**	**11 (48)**	**11 (24)**	**9 (82)**
Stimulant	14 (23)	4 (29)	8 (17)	4 (50)
Metformin	10 (17)	6 (60)	4 (9)	2 (50)
Topiramate	10 (17)	5 (50)	2 (4)	1 (50)
GLP-1 receptor agonist	5 (8)	2 (40)	1 (2)	1 (100)
Oxytocin	5 (8)	2 (40)	2 (4)	2 (100)
Naltrexone	2 (3)	1 (50)	2 (4)	2 (100)
Phentermine	0 (0)	–	1 (2)	1 (100)

The proportions of caregivers who reported trialing given strategies for weight management are shown, stratified by current obesity status. Of those who had tried a given strategy, we also reported the proportion who found the strategy helpful. The bold text designates the statistics corresponding to the respondent's perception of benefit for any intervention within the overall category, that is, Dietary Treatment, Exercise, or Medication.

### Peer Relationships

**Figure 1:**
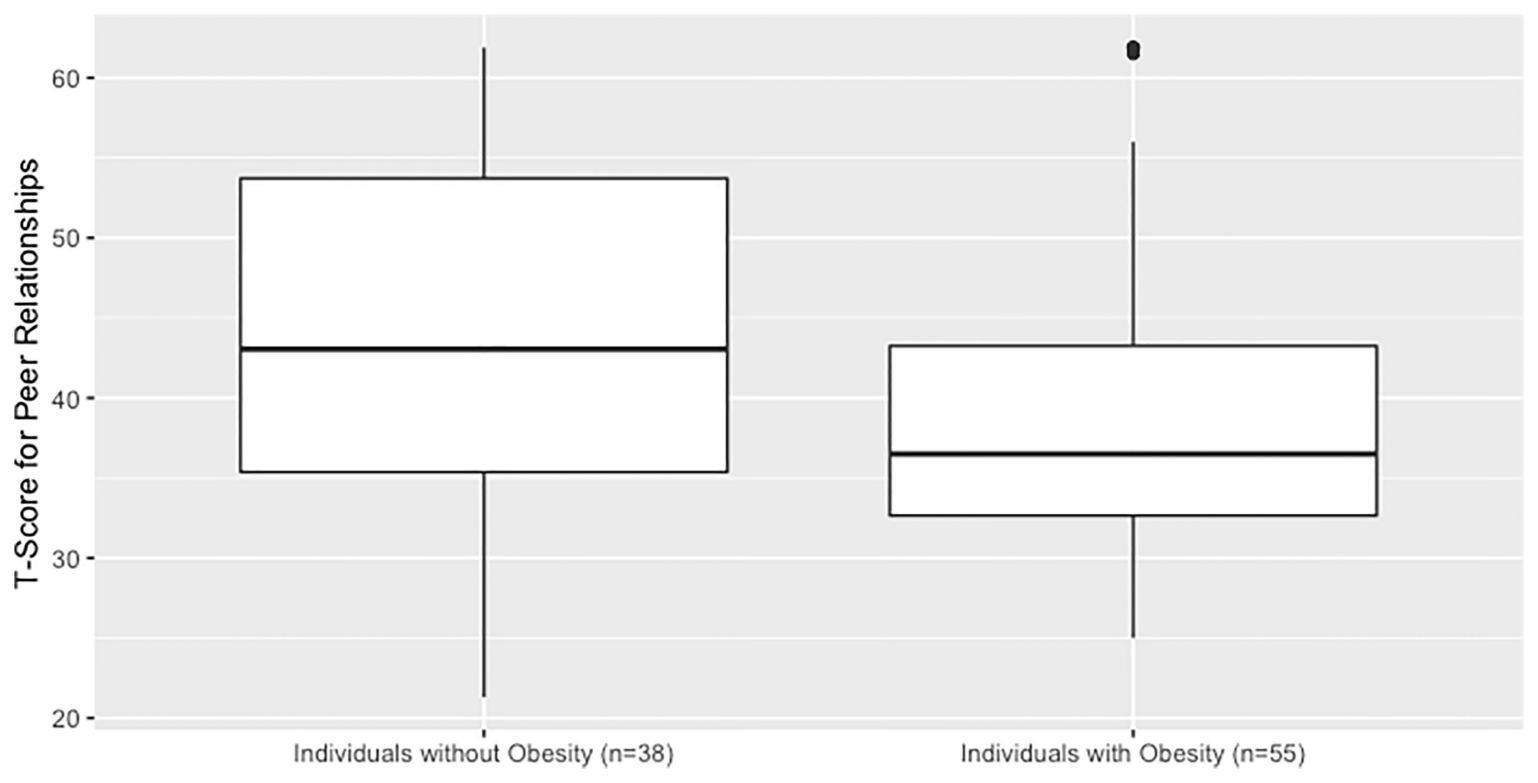
Social function in children and adolescents with craniopharyngioma without vs. with hypothalamic obesity. PROMIS parent-proxy measure of pediatric peer relationships. Caregivers completed a validated questionnaire which generates a quantitative T-score of their child’s social function. The questionnaire is designed to have a reference population mean of 50 and SD of 10, and a higher value corresponds to better social function.

The mean caregiver-reported T-score for craniopharyngioma survivors was 41 (SD 11), lower than the standardized mean of 50 (SD 10) in a reference population (p<0.001). Using two sample t-test, individuals with obesity had lower PROMIS scores than those without obesity, 44 versus 39 with a between-group difference of 5, 95% CI, 1-10, p = 0.02 (**Figure 1**). Older age at the time of the assessment was associated with lower T-Score and thus worse social function (p < 0.01). Females had marginally lower PROMIS T-Scores compared to males (38 versus 42, between-group difference of 4, 95% CI -8-0, p = 0.05). Individuals who had either photon or proton exposure scored lower than those without radiation exposure (43 versus 38, between-group difference of 5, 95% CI 1-9, p = 0.02). Individuals with any radiation exposure were more likely to have obesity than those without radiation exposure. In a linear regression model that included all of these variables, only age and obesity status were associated with PROMIS scores (see **Supplementary Table 4**).

### Research Priorities

**Figure 2:**
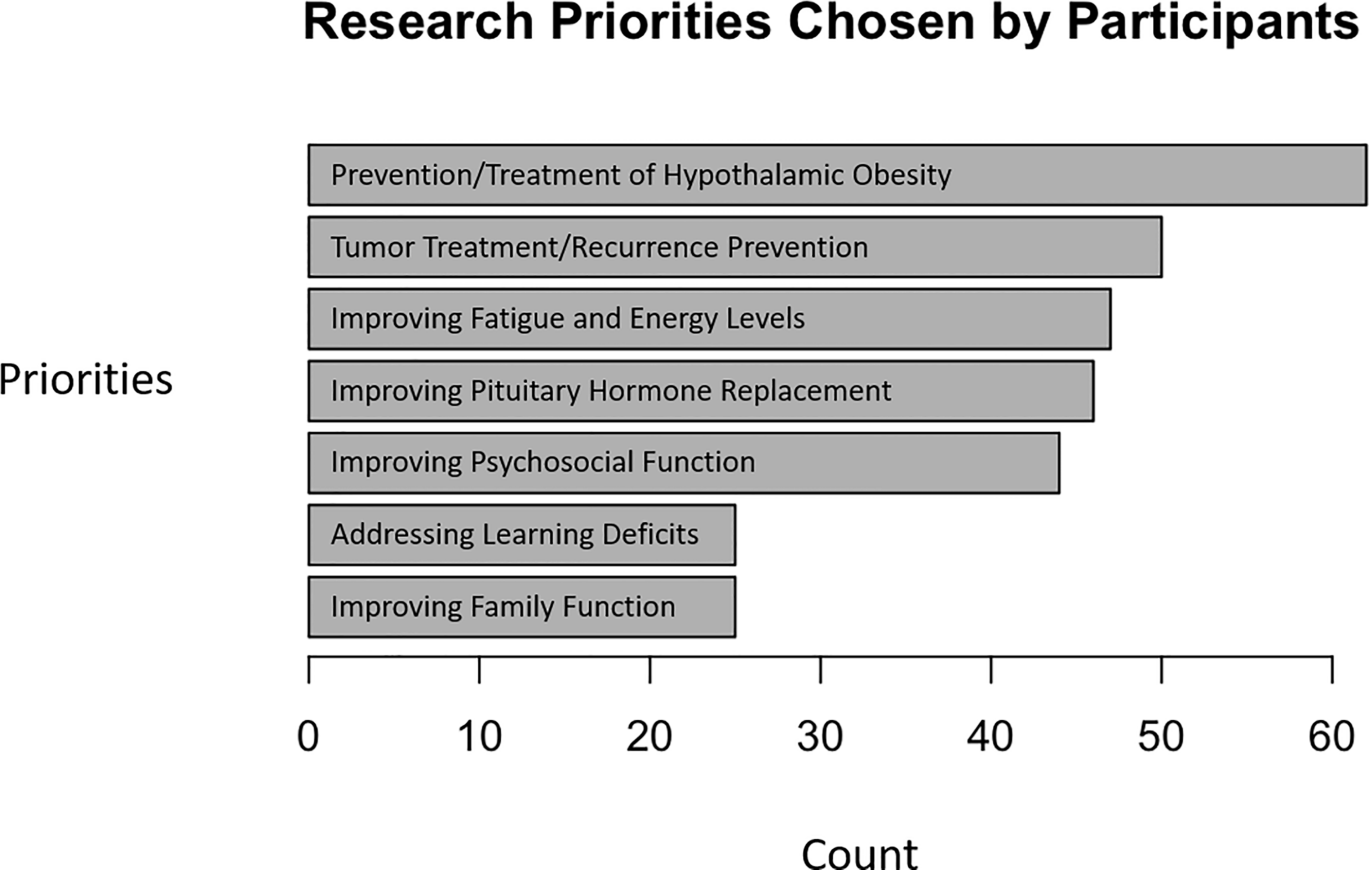
Research priorities of caregivers of children and adolescents with craniopharyngioma. Respondents indicated which topic(s) deserved attention in research related to craniopharyngioma.

The most frequently mentioned research priority was “improving treatments and prevention for hypothalamic obesity,” a response selected by 79% (62/78) of all respondents and 94% (44/47) of respondents whose child had obesity. In general, caregivers of individuals with obesity indicated more need for research in multiple domains (**Figure 2**).

## Discussion

Although survival is generally excellent in childhood craniopharyngioma, other sequelae, most notably hypothalamic obesity, can lead to excess morbidity and mortality and impaired quality of life for individuals and families (6, 7). In this study, we gathered patient-level details about how individuals and families respond to the challenges of hypothalamic obesity. From this work, we hope to develop interventions that are both more effective and more responsive to individual and family needs and priorities.

Our survey had several key findings. First, a minority of pediatric patients had trialed medication for weight loss even though most found lifestyle modification inadequate. We speculate that this low rate of medication use may be because the available medications (e.g., stimulants, metformin, topiramate) all have limitations with respect to efficacy, both as reported in the literature and by our survey respondents (17–23). Notably this study was completed prior to FDA approval of a GLP-1 receptor agonist (liraglutide) for obesity in adolescents as young as 12 years of age. Also, although there is some evidence of efficacy in the overall small number of pediatric patients treated with bariatric surgery, we did not focus on this option in our survey (24). The role for bariatric surgery and the expected course deserves additional attention in this cohort, in particular its role for addressing comorbidities.

As expected, a substantial proportion of individuals with hypothalamic obesity had tried modifying their diet and/or exercise for weight management. Just over half of individuals with obesity found these lifestyle modifications at least somewhat helpful. In individuals with both “common” and hypothalamic obesity, lifestyle modification alone can be insufficient to achieve weight loss, though can have many benefits for overall metabolic and mental health and are rightly part of any comprehensive weight loss intervention strategy (25, 26). There is some anecdotal evidence for protein-sparing modified fasts and low-carbohydrate diets in individuals with hypothalamic obesity, which is consistent with reports in our cohort that lower-carbohydrate approaches tended to be helpful (27). Most likely, nutrition and exercise are effective in maximizing the extent of the weight loss response to pharmacologic interventions and ensuring the sustainability of that response. Also, critically, as in “common” obesity, nutrition and exercise likely play a large role in preventing or mitigating metabolic sequelae of hypothalamic obesity, including fatty liver disease and type 2 diabetes mellitus (28, 29).

One surprising finding was that many individuals without obesity were incorporating lifestyle changes towards healthy eating and exercise. Individuals without obesity found that their lifestyle modification efforts seemed more helpful for weight management than their counterparts with obesity, perhaps reflecting their milder phenotype (i.e., lower degree of metabolic disruption), different background polygenic risk for obesity, and/or better alignment between the intervention and their needs. Our findings suggest that the work required to prevent hypothalamic obesity in some at-risk individuals may be under-recognized.

Especially in the absence of clear consensus guidelines for management of hypothalamic obesity, many pediatric endocrinologists may prefer to focus first on optimizing hormone replacement. There is some evidence supporting the benefits of judicious pituitary hormone replacement. For example, most practitioners will use the lowest dose of hydrocortisone that effectively manages symptoms of adrenal insufficiency in an attempt to avoid excess glucocorticoid related weight gain. At least one previous study indicates that adipocyte cortisol metabolism may favor cortisol accumulation in individuals with hypothalamic obesity, with excess cortisol leading to visceral fat accumulation (30). In regard to thyroid replacement, many experts endorse keeping free T4 in the upper part of normal range. And while there is no clear evidence supporting altering therapy with T3 monotherapy in hypothalamic obesity, the option of a trial of combined LT4/T3 has been explored in adult patients not satisfied with LT4 alone and may be worth considering in children as well (31, 32) as seen in a small subset of the patients in this study. However, there was no clear difference seen in glucocorticoid replacement or in thyroid replacement in our study between individuals with and without obesity. What we did find was that although 83% of caregivers endorsed a diagnosis of growth hormone deficiency, only 39% reported receiving treatment. Evidence suggests growth hormone initiation in childhood may improve BMI trajectory, fatigue, and emotional health in individuals with craniopharyngioma (33). In adults, weekly growth hormone formulation has been approved based on its ability to decrease truncal fat (34). It is important to emphasize the value of individualization of growth hormone treatment recommendations. The potential risk/benefit balance of growth hormone treatment in this cohort may be worth revisiting to avoid under-treatment and missing potential health benefits.

Ours is one of a few studies to evaluate peer relationships in survivors of childhood craniopharyngioma. Our findings indicate substantially decreased quality of peer relationships in youth with craniopharyngioma, with mean score from the current cohort one standard deviation below the mean for normative samples and published data for survivors of other childhood brain tumors (35). Of note, only 4% of the sample in the Lai et al. study included those with a craniopharyngioma, and the peer relationship scale used in that study was the self-reported scale with an observed mean of 50 (SD 11). Other studies have looked at peer relationships using the PROMIS instrument in otherwise healthy children with obesity, without craniopharyngioma. We posit that craniopharyngioma and obesity have additive adverse effects on social function. Selewski et al. (36) found that children with obesity (BMI > 99th percentile) had an average T score of 46.1 (SD 9.0) (36). This score is higher than our average population T-score of 41 (SD 11) and higher than the average T score of 39 among our participants with obesity. We acknowledge that we were unable to verify BMI in our survey population as obesity status was caregiver-reported. Future studies in survivors of pediatric brain tumors should evaluate the correspondence between self- and parent-report. We also found that both older age and obesity were independent risk factors for worse scores on parent report of peer relationships. While female sex and radiation treatment were associated with lower scores in univariate analyses, these associations were attenuated in the linear model that also included age and obesity. Unfortunately, pediatric obesity carries a social stigma of which children and adolescents may become more aware as they become older (37). Consistent with our findings, previous studies also have shown higher depression scores in those with severe forms of “common” obesity (BMI > 99th percentile) compared to children with BMI 85th-99th percentile (36). Prior studies also identified impairments in social cognition in survivors of childhood craniopharyngioma, including difficulties correctly interpreting tone of voice and others’ mental states that may underlie our findings (38–41). Given the importance of social relationships to youth mental health, these findings are highly concerning for this already at-risk population and worthy of additional investigation for factors impacting social functioning and opportunities to intervene (39, 40).

Our study has several strengths and limitations. The use of a web-based survey without collection of protected health information may have removed barriers to participating for many respondents. At the same time, our results may be most reflective of the subset of caregivers who have internet access and subscribe to the patient advocacy website and social media groups where links were posted. In addition, we did not collect demographic information regarding the caregivers who filled out the survey, such as the education level, ethnicity, and profession of the caregivers that could have impacted responses and led to a population bias. It is possible that the online format of the survey led to responses from more educated and well-resourced families, though it is also possible that some respondents preferred the anonymous format. We also did not collect information regarding the race of respondents, so we are not able to evaluate for disparities in care related to systemic racism and other factors that have been noted previously (1). This is particularly relevant because prior studies have indicated an increased risk of and decreased survival after craniopharyngioma in individuals identifying as African American, so future studies should be informed by best practices on collecting data regarding disparities in care (1, 42).

To shed light on the question of the generalizability of our findings, we compared the survey population to a cohort of patients with craniopharyngioma followed at our institution, The Children’s Hospital of Philadelphia. We found similar ages of diagnosis, male predominance, and similar rates of obesity after craniopharyngioma treatment. We did find difference in pre-craniopharyngioma rates of obesity. Specifically, 28% patients followed at CHOP had obesity (based on BMI Z-score) prior to diagnosis versus a pre-diagnosis obesity rate of 5.7% in the survey. One possibility is that caregivers may underestimate the degree of obesity (43). Another possibility is that the pre-diagnosis obesity rate in craniopharyngioma at CHOP reflects the background rate of pediatric obesity in the U.S. (approximately 20%) that may be higher than in the other parts of the world in which survey respondents lived (44). Future studies will benefit from taking additional measures to ensure that the data reflect the educational, socioeconomic, and racial/ethnic diversity of individuals with craniopharyngioma and their families.

Another limitation of this survey was we did not have a means to independently verify the self-reported medical history information using the medical records. Also, some participants elected not to complete all survey components, in particular the detailed medication review, which required additional time. We found the participant characteristics to be similar between the group that filled out each survey component and the broader demographic survey (n=106), see **Supplementary Table 1**. While only 8.7% of caregivers reported their children being on no medications, 35% of caregivers did not complete the more time-consuming medication survey so we did not have the ability to align self-reports of hormonal deficiency with medication approaches for treatment in all participants. However, in the subset of individuals who did complete both medical history and medication details, we did in general find good agreement as seen in **Supplementary Table 3**.

Despite these limitations, our survey provides additional evidence around current practices and supports continued research around treatment and quality of life in those with craniopharyngioma and hypothalamic obesity. Survivors of childhood craniopharyngioma take a large number of medications for pituitary insufficiency and other conditions; the apparently limited efficacy of medications for hypothalamic obesity adds frustration to this burden. We also found substantially decreased indices of social function in all children with craniopharyngioma, modestly more in individuals with obesity, which is consistent with previous work in survivors of other childhood brain tumors (35).

One unique aspect of our survey was that we explicitly asked caregivers about their research priorities. The most frequently mentioned research priority was around treating and preventing hypothalamic obesity. Notably, more respondents chose this research priority than prioritizing research on treatment and prevention of recurrence of craniopharyngioma itself. We speculate that this prioritization reflects the significant co-morbidities that come with surgery and radiation that are most often used to treat craniopharyngioma. Taken together, our findings highlight not only the challenges caregivers and individuals face, but also the value of collaboration between all stakeholders – patients, families, clinicians, and researchers – in developing research priorities in this condition.

## Data Availability Statement

Raw data supporting the conclusions of this article will be made available from the corresponding author (SM) on request. The data are not publicly available due to their containing clinical information that could be identifying in this rare disorder.

## Ethics Statement

Ethical review and approval was not required for the study on human participants in accordance with the local legislation and institutional requirements. Written informed consent for participation was not required for this study in accordance with the national legislation and the institutional requirements.

## Author Contributions

MC and JC performed the statistical analysis and wrote the first draft of the manuscript. LC organized the database and performed initial analysis. SM contributed to conception and design of the study and supervised MC, JC, and LC. All authors contributed to critical manuscript revisions, and both read and approved the final submitted version.

## Funding

This work was supported by the Children’s Hospital of Philadelphia, as well as Mike and Angela Finamore.

## Conflict of Interest

The authors declare that the research was conducted in the absence of any commercial or financial relationships that could be construed as a potential conflict of interest.

## Publisher’s Note

All claims expressed in this article are solely those of the authors and do not necessarily represent those of their affiliated organizations, or those of the publisher, the editors and the reviewers. Any product that may be evaluated in this article, or claim that may be made by its manufacturer, is not guaranteed or endorsed by the publisher.
