# Supplementary material for: A Survey of Patient-Relevant Outcomes in Pediatric Craniopharyngioma: Focus on Hypothalamic Obesity
Source: Front Endocrinol (Lausanne). 2022 May 9;13:876770. doi: 10.3389/fendo.2022.876770 (PMC9124861; doi:10.3389/fendo.2022.876770)
Supplement: Supplementary file 1 [file DataSheet_1.pdf]

Psychosocial function and metabolism in children with hypothalamic/pituitary brain tumors

Researchers at the Children's Hospital of Philadelphia are planning new research related to the psychosocial and metabolic complications of childhood brain tumors.

This questionnaire is designed to be completed by adult caregivers. The questions focus on children under 18 years of age who currently have brain tumors affecting the hypothalamus and/or pituitary, and/or who have received treatment in the past for brain tumors affecting the hypothalamus and/or pituitary.

The information you provide will help to guide these efforts.

Thank you in advance for partnering with us in identifying priorities for research!

Please note: These questionnaires are not intended to diagnose or treat any clinical conditions. Please raise any related concerns with your healthcare provider. Also, please do not include any identifying information in your responses.

By completing this survey, you are giving consent to participate.

If you have question, please contact the study team, Ms. Kristin Wade (wadekl@email.chop.edu) or Ms. Anna Dedio (dedioa@email.chop.edu).

There are 4 survey pages, we estimate this will take between ~10 and 15 minutes to complete.

Are you 18 years of age or older?

○ Yes
○ No

How old was your child at diagnosis (in years)?

____
((in years))

How old is your child now (in years)?

____
((in years))

Does your child have now (or did your child have in the past) a brain tumor affecting the hypothalamus and/or pituitary?

○ Yes
○ No

If your child does not have a brain tumor, what is your child's diagnosis?

____

What type of brain tumor does your child have (or did your child have in the past)?

○ Craniopharyngioma
○ Astrocytoma
○ Ependymoma
○ Medulloblastoma
○ Other
○ Do not know

What is your child's sex?

○ Female
○ Male
○ Other

Has your child received any of the following interventions (indicate all received)?	☐ Neurosurgery (trans-sphenoidal/endonasal)<br>☐ Neurosurgery (open/craniotomy)<br>☐ Neurosurgery (cyst drainage)<br>☐ Radiation (not proton beam) therapy<br>☐ Proton beam therapy<br>☐ Chemotherapy<br>☐ Other (please specify)
What other treatment did your child receive?	__________________________
Does your child currently have diabetes insipidus?	◯ Yes<br>◯ No<br>◯ Unsure
Does your child currently have growth hormone deficiency?	◯ Yes<br>◯ No<br>◯ Unsure
Does your child currently have adrenal insufficiency?	◯ Yes<br>◯ No<br>◯ Unsure
Does your child currently have hypothyroidism?	◯ Yes<br>◯ No<br>◯ Unsure
Does your child have a history of precocious (early) puberty?	◯ Yes<br>◯ No<br>◯ Unsure
Does your child currently have hypogonadism (low estrogen for girls, low testosterone for boys)?	◯ Yes<br>◯ No<br>◯ Unsure<br>◯ Not applicable (too young to know)
Is your child currently obese (usually defined as a body mass index, BMI, greater than or equal to the 95th percentile for age and sex)?	◯ No<br>◯ Yes<br>◯ Unsure
Has your child ever been obese (usually defined as a body mass index, BMI, greater than or equal to the 95th percentile for age and sex)?	◯ No<br>◯ Yes<br>◯ Unsure
Prior to brain tumor diagnosis, was your child obese (usually defined as a body mass index, BMI, greater than or equal to the 95th percentile for age and sex)?	◯ No<br>◯ Yes<br>◯ Unsure

Which of the following (indicate as many as apply) has your child tried to manage excess weight gain?	☐ diet - limiting calories<br>☐ diet - limiting carbohydrates<br>☐ diet - other (please specify)<br>☐ exercise - independent<br>☐ exercise - supervised (e.g., with a trainer or coach)<br>☐ medication - stimulant (e.g., methylphenidate, dextro-amphetamine)<br>☐ medication - Metformin<br>☐ medication - GLP1 agonist (e.g., exenatide, liraglutide, semaglutide)<br>☐ medication - topirimate<br>☐ medication - naltrexone<br>☐ medication - oxytocin<br>☐ medication - lorcaserin<br>☐ medication - other (please specify)<br>☐ not sure<br>☐ not applicable - my child has never tried to manage excess weight gain
What other diet was tried to manage excess weight gain?	__________________________________
What other medication was tried to manage excess weight gain?	__________________________________
Which of the following (indicate as many as apply) have you found helpful to manage excess weight gain?	☐ diet - limiting calories<br>☐ diet - limiting carbohydrates<br>☐ diet - other (please specify)<br>☐ exercise - independent<br>☐ exercise - supervised (e.g., with a trainer or coach)<br>☐ medication - stimulant (e.g., methylphenidate, dextro-amphetamine)<br>☐ medication - Metformin<br>☐ medication - GLP1 agonist (e.g., exenatide, liraglutide, semaglutide)<br>☐ medication - topirimate<br>☐ medication - naltrexone<br>☐ medication - oxytocin<br>☐ medication - lorcaserin<br>☐ medication - other (please specify)<br>☐ not sure<br>☐ not applicable - my child has never tried to manage excess weight gain
What other diet was helpful to manage excess weight gain?	__________________________________
What other medication was helpful to to manage excess weight gain?	__________________________________

Peer relationships

The quality of peer relationships is important for all children, in particular those affected by medical conditions. We are using a brief parent-reported instrument to get a "snapshot" of these relationships.

This questionnaire was developed and validated by the National Institutes of Health Patient-Reported Outcome Measurement Information System (PROMIS). It is an adult caregiver-reported assessment of children's peer relationships.

Please respond to each question or statement by marking one box per row.

In the past 7 days...

		Never	Almost Never	Sometimes	Often	Almost Always
1	My child felt accepted by other kids his/her age.	○	○	○	○	○
2	My child was able to count on his/her friends.	○	○	○	○	○
3	My child was good at making friends.	○	○	○	○	○
4	My child and his/her friends helped each other out.	○	○	○	○	○
5	Other kids wanted to be my child's friend.	○	○	○	○	○
6	Other kids wanted to be with my child.	○	○	○	○	○
7	Other kids wanted to talk to my child.	○	○	○	○	○

Research priorities

Researchers at the Children's Hospital of Philadelphia are planning new research related to the psychosocial and metabolic complications of childhood brain tumors.

This questionnaire is designed to be completed by the adult caregivers of children under 18 years of age with brain tumors affecting the hypothalamus and/or pituitary.

The information you provide will help to guide these efforts.

Thank you in advance for partnering with us in identifying priorities for research!

Please note: These questionnaires are not intended to diagnose or treat any clinical conditions. Please raise any related concerns with your healthcare provider. Also, please do not include any identifying information in your responses.

What are the most important research questions related to your child's hypothalamic/pituitary brain tumor (select all that apply)?	☐ How can my child's tumor be more effectively treated and/or how can recurrence be prevented?<br>☐ How can my child's psychosocial function be improved?<br>☐ How can my child's fatigue/energy level be improved?<br>☐ How can my child's pituitary hormonal replacement be improved?<br>☐ How can hypothalamic obesity be prevented or treated?<br>☐ How can my child's learning deficits be addressed?<br>☐ How can our family's function be improved?<br>☐ Other (please specify)
What are other important areas/questions for future research?	________________________
Has your child ever participated in a research study?	◯ Yes<br>◯ No
Is there any other information you think we should know in planning for future research? (As a reminder, please do not include personal or identifying health information here.)	______________________________

Medications & supplements

It is helpful in planning for future studies to know details related to medications and supplements that children are taking.

However, inputting medication and supplement details can be time-consuming! If you prefer, you can skip this section by scrolling to the bottom and clicking "Submit", and finish by sharing your thoughts regarding priorities for research.

Thank you!

Is your child taking any medications? Please also include any nutritional supplements.	◯ Yes ◯ No
Prescription (RX) or over-the-counter (OTC)?	◯ RX<br>◯ OTC
Medication Name	______________________
[cm_med1] Indication	______________________
Dose Unit for [cm_med1]	◯ microgram (mcg)<br>◯ milligram (mg)<br>◯ gram (g)<br>◯ international units (IU)<br>◯ other<br>◯ unknown
If other, please specify dose unit	______________________
[cm_med1] Dose	______________________
Dose Frequency for [cm_med1]	◯ Q1D (daily)<br>◯ BID (twice daily)<br>◯ TID (three times a day)<br>◯ QD (four times a day)<br>◯ 4 - 6 hrs<br>◯ 6 - 8 hrs<br>◯ PRN (as needed)<br>◯ Other<br>◯ Unknown
If other, please specify frequency	______________________
Is medication as needed (PRN)?	◯ Yes<br>◯ No
[cm_med1] Route	◯ PO (by mouth)<br>◯ SC (subcutaneous)<br>◯ IN (intranasal)<br>◯ IM (intramuscular)<br>◯ TOP (topical)<br>◯ Other

If other, please specify route

____

Additional comments about [cm_med1] use

____

Next Medication

Prescription (RX) or over-the-counter (OTC)?

- ◯ RX
- ◯ OTC

Medication Name

____

[cm_med2] Indication

____

Dose Unit for [cm_med2]

- ◯ microgram (mcg)
- ◯ milligram (mg)
- ◯ gram (g)
- ◯ international units (IU)
- ◯ other
- ◯ unknown

If other, please specify dose unit

____

[cm_med2] Dose

____

Dose Frequency for [cm_med2]

- ◯ Q1D (daily)
- ◯ BID (twice daily)
- ◯ TID (three times a day)
- ◯ QD (four times a day)
- ◯ 4 - 6 hrs
- ◯ 6 - 8 hrs
- ◯ PRN (as needed)
- ◯ Other
- ◯ Unknown

If other, please specify frequency

____

Is medication as needed (PRN)?

- ◯ Yes
- ◯ No

[cm_med2] Route

- ◯ PO (by mouth)
- ◯ SC (subcutaneous)
- ◯ IN (intranasal)
- ◯ IM (intramuscular)
- ◯ TOP (topical)
- ◯ Other

If other, please specify route

____

Additional comments about [cm_med2] use

____

Next Medication

Prescription (RX) or over-the-counter (OTC)?

- ◯ RX
- ◯ OTC

Medication Name

____

[cm_med3] Indication

____

Dose Unit for [cm_med3]

- ◯ microgram (mcg)
- ◯ milligram (mg)
- ◯ gram (g)
- ◯ international units (IU)
- ◯ other
- ◯ unknown

If other, please specify dose unit

____

[cm_med3] Dose

____

Dose Frequency for [cm_med3]

- ◯ Q1D (daily)
- ◯ BID (twice daily)
- ◯ TID (three times a day)
- ◯ QD (four times a day)
- ◯ 4 - 6 hrs
- ◯ 6 - 8 hrs
- ◯ PRN (as needed)
- ◯ Other
- ◯ Unknown

If other, please specify frequency

____

Is medication as needed (PRN)?

- ◯ Yes
- ◯ No

[cm_med3] Route

- ◯ PO (by mouth)
- ◯ SC (subcutaneous)
- ◯ IN (intranasal)
- ◯ IM (intramuscular)
- ◯ TOP (topical)
- ◯ Other

If other, please specify route

____

Additional comments about [cm_med3] use

____

Next Medication

Prescription (RX) or over-the-counter (OTC)?	◯ RX<br>◯ OTC
Medication Name	__________________
[cm_med4] Indication	__________________
Dose Unit for [cm_med4]	◯ microgram (mcg)<br>◯ milligram (mg)<br>◯ gram (g)<br>◯ international units (IU)<br>◯ other<br>◯ unknown
If other, please specify dose unit	__________________
[cm_med4] Dose	__________________
Dose Frequency for [cm_med4]	◯ Q1D (daily)<br>◯ BID (twice daily)<br>◯ TID (three times a day)<br>◯ QD (four times a day)<br>◯ 4 - 6 hrs<br>◯ 6 - 8 hrs<br>◯ PRN (as needed)<br>◯ Other<br>◯ Unknown
If other, please specify frequency	__________________
Is medication as needed (PRN)?	◯ Yes<br>◯ No
[cm_med4] Route	◯ PO (by mouth)<br>◯ SC (subcutaneous)<br>◯ IN (intranasal)<br>◯ IM (intramuscular)<br>◯ TOP (topical)<br>◯ Other
If other, please specify route	__________________
Additional comments about [cm_med4] use	______________________

Next Medication

Prescription (RX) or over-the-counter (OTC)?	◯ RX<br>◯ OTC
Medication Name	__________________________
[cm_med5] Indication	__________________________
Dose Unit for [cm_med5]	◯ microgram (mcg)<br>◯ milligram (mg)<br>◯ gram (g)<br>◯ international units (IU)<br>◯ other<br>◯ unknown
If other, please specify dose unit	__________________________
[cm_med5] Dose	__________________________
Dose Frequency for [cm_med5]	◯ Q1D (daily)<br>◯ BID (twice daily)<br>◯ TID (three times a day)<br>◯ QD (four times a day)<br>◯ 4 - 6 hrs<br>◯ 6 - 8 hrs<br>◯ PRN (as needed)<br>◯ Other<br>◯ Unknown
If other, please specify frequency	__________________________
Is medication as needed (PRN)?	◯ Yes<br>◯ No
[cm_med5] Route	◯ PO (by mouth)<br>◯ SC (subcutaneous)<br>◯ IN (intranasal)<br>◯ IM (intramuscular)<br>◯ TOP (topical)<br>◯ Other
If other, please specify route	__________________________
Additional comments about [cm_med5] use	__________________________________

Next Medication

Prescription (RX) or over-the-counter (OTC)?	◯ RX<br>◯ OTC
Medication Name	______________________
[cm_med6] Indication	______________________
Dose Unit for [cm_med6]	◯ microgram (mcg)<br>◯ milligram (mg)<br>◯ gram (g)<br>◯ international units (IU)<br>◯ other<br>◯ unknown
If other, please specify dose unit	______________________
[cm_med6] Dose	______________________
Dose Frequency for [cm_med6]	◯ Q1D (daily)<br>◯ BID (twice daily)<br>◯ TID (three times a day)<br>◯ QD (four times a day)<br>◯ 4 - 6 hrs<br>◯ 6 - 8 hrs<br>◯ PRN (as needed)<br>◯ Other<br>◯ Unknown
If other, please specify frequency	______________________
Is medication as needed (PRN)?	◯ Yes<br>◯ No
[cm_med6] Route	◯ PO (by mouth)<br>◯ SC (subcutaneous)<br>◯ IN (intranasal)<br>◯ IM (intramuscular)<br>◯ TOP (topical)<br>◯ Other
If other, please specify route	______________________
Additional comments about [cm_med6] use	____________________________

Next Medication

Prescription (RX) or over-the-counter (OTC)?	◯ RX<br>◯ OTC
Medication Name	____________________
[cm_med7] Indication	____________________
Dose Unit for [cm_med7]	◯ microgram (mcg)<br>◯ milligram (mg)<br>◯ gram (g)<br>◯ international units (IU)<br>◯ other<br>◯ unknown
If other, please specify dose unit	____________________
[cm_med7] Dose	____________________
Dose Frequency for [cm_med7]	◯ Q1D (daily)<br>◯ BID (twice daily)<br>◯ TID (three times a day)<br>◯ QD (four times a day)<br>◯ 4 - 6 hrs<br>◯ 6 - 8 hrs<br>◯ PRN (as needed)<br>◯ Other<br>◯ Unknown
If other, please specify frequency	____________________
Is medication as needed (PRN)?	◯ Yes<br>◯ No
[cm_med7] Route	◯ PO (by mouth)<br>◯ SC (subcutaneous)<br>◯ IN (intranasal)<br>◯ IM (intramuscular)<br>◯ TOP (topical)<br>◯ Other
If other, please specify route	____________________
Additional comments about [cm_med7] use	____________________

Next Medication

Prescription (RX) or over-the-counter (OTC)?	◯ RX<br>◯ OTC
Medication Name	__________________________
[cm_med8] Indication	__________________________
Dose Unit for [cm_med8]	◯ microgram (mcg)<br>◯ milligram (mg)<br>◯ gram (g)<br>◯ international units (IU)<br>◯ other<br>◯ unknown
If other, please specify dose unit	__________________________
[cm_med8] Dose	__________________________
Dose Frequency for [cm_med8]	◯ Q1D (daily)<br>◯ BID (twice daily)<br>◯ TID (three times a day)<br>◯ QD (four times a day)<br>◯ 4 - 6 hrs<br>◯ 6 - 8 hrs<br>◯ PRN (as needed)<br>◯ Other<br>◯ Unknown
If other, please specify frequency	__________________________
Is medication as needed (PRN)?	◯ Yes<br>◯ No
[cm_med8] Route	◯ PO (by mouth)<br>◯ SC (subcutaneous)<br>◯ IN (intranasal)<br>◯ IM (intramuscular)<br>◯ TOP (topical)<br>◯ Other
If other, please specify route	__________________________
Additional comments about [cm_med8] use	________________________________

Next Medication

Prescription (RX) or over-the-counter (OTC)?	◯ RX<br>◯ OTC
Medication Name	__________________________________
[cm_med9] Indication	__________________________________
Dose Unit for [cm_med9]	◯ microgram (mcg)<br>◯ milligram (mg)<br>◯ gram (g)<br>◯ international units (IU)<br>◯ other<br>◯ unknown
If other, please specify dose unit	__________________________________
[cm_med9] Dose	__________________________________
Dose Frequency for [cm_med9]	◯ Q1D (daily)<br>◯ BID (twice daily)<br>◯ TID (three times a day)<br>◯ QD (four times a day)<br>◯ 4 - 6 hrs<br>◯ 6 - 8 hrs<br>◯ PRN (as needed)<br>◯ Other<br>◯ Unknown
If other, please specify frequency	__________________________________
Is medication as needed (PRN)?	◯ Yes<br>◯ No
[cm_med9] Route	◯ PO (by mouth)<br>◯ SC (subcutaneous)<br>◯ IN (intranasal)<br>◯ IM (intramuscular)<br>◯ TOP (topical)<br>◯ Other
If other, please specify route	__________________________________
Additional comments about [cm_med9] use	____

Next Medication

Prescription (RX) or over-the-counter (OTC)?	◯ RX<br>◯ OTC
Medication Name	______________________
[cm_med10] Indication	______________________
Dose Unit for [cm_med10]	◯ microgram (mcg)<br>◯ milligram (mg)<br>◯ gram (g)<br>◯ international units (IU)<br>◯ other<br>◯ unknown
If other, please specify dose unit	______________________
[cm_med10] Dose	______________________
Dose Frequency for [cm_med10]	◯ Q1D (daily)<br>◯ BID (twice daily)<br>◯ TID (three times a day)<br>◯ QD (four times a day)<br>◯ 4 - 6 hrs<br>◯ 6 - 8 hrs<br>◯ PRN (as needed)<br>◯ Other<br>◯ Unknown
If other, please specify frequency	______________________
Is medication as needed (PRN)?	◯ Yes<br>◯ No
[cm_med10] Route	◯ PO (by mouth)<br>◯ SC (subcutaneous)<br>◯ IN (intranasal)<br>◯ IM (intramuscular)<br>◯ TOP (topical)<br>◯ Other
If other, please specify route	______________________
Additional comments about [cm_med10] use	____________________________

Next Medication

Prescription (RX) or over-the-counter (OTC)?	◯ RX<br>◯ OTC
Medication Name	______________________
[cm_med11] Indication	______________________
Dose Unit for [cm_med11]	◯ microgram (mcg)<br>◯ milligram (mg)<br>◯ gram (g)<br>◯ international units (IU)<br>◯ other<br>◯ unknown
If other, please specify dose unit	______________________
[cm_med11] Dose	______________________
Dose Frequency for [cm_med11]	◯ Q1D (daily)<br>◯ BID (twice daily)<br>◯ TID (three times a day)<br>◯ QD (four times a day)<br>◯ 4 - 6 hrs<br>◯ 6 - 8 hrs<br>◯ PRN (as needed)<br>◯ Other<br>◯ Unknown
If other, please specify frequency	______________________
Is medication as needed (PRN)?	◯ Yes<br>◯ No
[cm_med11] Route	◯ PO (by mouth)<br>◯ SC (subcutaneous)<br>◯ IN (intranasal)<br>◯ IM (intramuscular)<br>◯ TOP (topical)<br>◯ Other
If other, please specify route	______________________
Additional comments about [cm_med11] use	____________________________

Next Medication

Prescription (RX) or over-the-counter (OTC)?	◯ RX<br>◯ OTC
Medication Name	__________________
[cm_med12] Indication	__________________
Dose Unit for [cm_med12]	◯ microgram (mcg)<br>◯ milligram (mg)<br>◯ gram (g)<br>◯ international units (IU)<br>◯ other<br>◯ unknown
If other, please specify dose unit	__________________
[cm_med12] Dose	__________________
Dose Frequency for [cm_med12]	◯ Q1D (daily)<br>◯ BID (twice daily)<br>◯ TID (three times a day)<br>◯ QD (four times a day)<br>◯ 4 - 6 hrs<br>◯ 6 - 8 hrs<br>◯ PRN (as needed)<br>◯ Other<br>◯ Unknown
If other, please specify frequency	__________________
Is medication as needed (PRN)?	◯ Yes<br>◯ No
[cm_med12] Route	◯ PO (by mouth)<br>◯ SC (subcutaneous)<br>◯ IN (intranasal)<br>◯ IM (intramuscular)<br>◯ TOP (topical)<br>◯ Other
If other, please specify route	__________________
Additional comments about [cm_med12] use	______________________

Next Medication

Prescription (RX) or over-the-counter (OTC)?	◯ RX<br>◯ OTC
Medication Name	__________________
[cm_med13] Indication	__________________
Dose Unit for [cm_med13]	◯ microgram (mcg)<br>◯ milligram (mg)<br>◯ gram (g)<br>◯ international units (IU)<br>◯ other<br>◯ unknown
If other, please specify dose unit	__________________
[cm_med13] Dose	__________________
Dose Frequency for [cm_med13]	◯ Q1D (daily)<br>◯ BID (twice daily)<br>◯ TID (three times a day)<br>◯ QD (four times a day)<br>◯ 4 - 6 hrs<br>◯ 6 - 8 hrs<br>◯ PRN (as needed)<br>◯ Other<br>◯ Unknown
If other, please specify frequency	__________________
Is medication as needed (PRN)?	◯ Yes<br>◯ No
[cm_med13] Route	◯ PO (by mouth)<br>◯ SC (subcutaneous)<br>◯ IN (intranasal)<br>◯ IM (intramuscular)<br>◯ TOP (topical)<br>◯ Other
If other, please specify route	__________________
Additional comments about [cm_med13] use	______________________
Have there been any changes to this medication since the last visit?	◯ Yes<br>◯ No<br>◯ N/A

Next Medication

Prescription (RX) or over-the-counter (OTC)?	◯ RX<br>◯ OTC
Medication Name	________________
[cm_med14] Indication	________________
Dose Unit for [cm_med14]	◯ microgram (mcg)<br>◯ milligram (mg)<br>◯ gram (g)<br>◯ international units (IU)<br>◯ other<br>◯ unknown
If other, please specify dose unit	________________
[cm_med14] Dose	________________
Dose Frequency for [cm_med14]	◯ Q1D (daily)<br>◯ BID (twice daily)<br>◯ TID (three times a day)<br>◯ QD (four times a day)<br>◯ 4 - 6 hrs<br>◯ 6 - 8 hrs<br>◯ PRN (as needed)<br>◯ Other<br>◯ Unknown
If other, please specify frequency	________________
Is medication as needed (PRN)?	◯ Yes<br>◯ No
[cm_med14] Route	◯ PO (by mouth)<br>◯ SC (subcutaneous)<br>◯ IN (intranasal)<br>◯ IM (intramuscular)<br>◯ TOP (topical)<br>◯ Other
If other, please specify route	________________
Additional comments about [cm_med14] use	________________

Next Medication

Prescription (RX) or over-the-counter (OTC)?	◯ RX<br>◯ OTC
Medication Name	__________________________
[cm_med15] Indication	__________________________
Dose Unit for [cm_med15]	◯ microgram (mcg)<br>◯ milligram (mg)<br>◯ gram (g)<br>◯ international units (IU)<br>◯ other<br>◯ unknown
If other, please specify dose unit	__________________________
[cm_med15] Dose	__________________________
Dose Frequency for [cm_med15]	◯ Q1D (daily)<br>◯ BID (twice daily)<br>◯ TID (three times a day)<br>◯ QD (four times a day)<br>◯ 4 - 6 hrs<br>◯ 6 - 8 hrs<br>◯ PRN (as needed)<br>◯ Other<br>◯ Unknown
If other, please specify frequency	__________________________
Is medication as needed (PRN)?	◯ Yes<br>◯ No
[cm_med15] Route	◯ PO (by mouth)<br>◯ SC (subcutaneous)<br>◯ IN (intranasal)<br>◯ IM (intramuscular)<br>◯ TOP (topical)<br>◯ Other
If other, please specify route	__________________________
Additional comments about [cm_med15] use	__________________________________

Next Medication

Prescription (RX) or over-the-counter (OTC)?	◯ RX<br>◯ OTC
Medication Name	______________________
[cm_med16] Indication	______________________
Dose Unit for [cm_med16]	◯ microgram (mcg)<br>◯ milligram (mg)<br>◯ gram (g)<br>◯ international units (IU)<br>◯ other<br>◯ unknown
If other, please specify dose unit	______________________
[cm_med16] Dose	______________________
Dose Frequency for [cm_med16]	◯ Q1D (daily)<br>◯ BID (twice daily)<br>◯ TID (three times a day)<br>◯ QD (four times a day)<br>◯ 4 - 6 hrs<br>◯ 6 - 8 hrs<br>◯ PRN (as needed)<br>◯ Other<br>◯ Unknown
If other, please specify frequency	______________________
Is medication as needed (PRN)?	◯ Yes<br>◯ No
[cm_med16] Route	◯ PO (by mouth)<br>◯ SC (subcutaneous)<br>◯ IN (intranasal)<br>◯ IM (intramuscular)<br>◯ TOP (topical)<br>◯ Other
If other, please specify route	______________________
Additional comments about [cm_med16] use	____________________________

Next Medication

Prescription (RX) or over-the-counter (OTC)?	◯ RX<br>◯ OTC
Medication Name	________________________
[cm_med17] Indication	________________________
Dose Unit for [cm_med17]	◯ microgram (mcg)<br>◯ milligram (mg)<br>◯ gram (g)<br>◯ international units (IU)<br>◯ other<br>◯ unknown
If other, please specify dose unit	________________________
[cm_med17] Dose	________________________
Dose Frequency for [cm_med17]	◯ Q1D (daily)<br>◯ BID (twice daily)<br>◯ TID (three times a day)<br>◯ QD (four times a day)<br>◯ 4 - 6 hrs<br>◯ 6 - 8 hrs<br>◯ PRN (as needed)<br>◯ Other<br>◯ Unknown
If other, please specify frequency	________________________
Is medication as needed (PRN)?	◯ Yes<br>◯ No
[cm_med17] Route	◯ PO (by mouth)<br>◯ SC (subcutaneous)<br>◯ IN (intranasal)<br>◯ IM (intramuscular)<br>◯ TOP (topical)<br>◯ Other
If other, please specify route	________________________
Additional comments about [cm_med17] use	______________________________
Have there been any changes to this medication since the last visit?	◯ Yes<br>◯ No<br>◯ N/A

Next Medication

Prescription (RX) or over-the-counter (OTC)?	◯ RX<br>◯ OTC
Medication Name	__________________________
[cm_med18] Indication	__________________________
Dose Unit for [cm_med18]	◯ microgram (mcg)<br>◯ milligram (mg)<br>◯ gram (g)<br>◯ international units (IU)<br>◯ other<br>◯ unknown
If other, please specify dose unit	__________________________
[cm_med18] Dose	__________________________
Dose Frequency for [cm_med18]	◯ Q1D (daily)<br>◯ BID (twice daily)<br>◯ TID (three times a day)<br>◯ QD (four times a day)<br>◯ 4 - 6 hrs<br>◯ 6 - 8 hrs<br>◯ PRN (as needed)<br>◯ Other<br>◯ Unknown
If other, please specify frequency	__________________________
Is medication as needed (PRN)?	◯ Yes<br>◯ No
[cm_med18] Route	◯ PO (by mouth)<br>◯ SC (subcutaneous)<br>◯ IN (intranasal)<br>◯ IM (intramuscular)<br>◯ TOP (topical)<br>◯ Other
If other, please specify route	__________________________
Additional comments about [cm_med18] use	________________________________

Next Medication

Prescription (RX) or over-the-counter (OTC)?	◯ RX<br>◯ OTC
Medication Name	__________________________________
[cm_med19] Indication	__________________________________
Dose Unit for [cm_med19]	◯ microgram (mcg)<br>◯ milligram (mg)<br>◯ gram (g)<br>◯ international units (IU)<br>◯ other<br>◯ unknown
If other, please specify dose unit	__________________________________
[cm_med19] Dose	__________________________________
Dose Frequency for [cm_med19]	◯ Q1D (daily)<br>◯ BID (twice daily)<br>◯ TID (three times a day)<br>◯ QD (four times a day)<br>◯ 4 - 6 hrs<br>◯ 6 - 8 hrs<br>◯ PRN (as needed)<br>◯ Other<br>◯ Unknown
If other, please specify frequency	__________________________________
Is medication as needed (PRN)?	◯ Yes<br>◯ No
[cm_med19] Route	◯ PO (by mouth)<br>◯ SC (subcutaneous)<br>◯ IN (intranasal)<br>◯ IM (intramuscular)<br>◯ TOP (topical)<br>◯ Other
If other, please specify route	__________________________________
Additional comments about [cm_med19] use	____

Next Medication

Prescription (RX) or over-the-counter (OTC)?	◯ RX<br>◯ OTC
Medication Name	__________________________
[cm_med20] Indication	__________________________
Dose Unit for [cm_med20]	◯ microgram (mcg)<br>◯ milligram (mg)<br>◯ gram (g)<br>◯ international units (IU)<br>◯ other<br>◯ unknown
If other, please specify dose unit	__________________________
[cm_med20] Dose	__________________________
Dose Frequency for [cm_med20]	◯ Q1D (daily)<br>◯ BID (twice daily)<br>◯ TID (three times a day)<br>◯ QD (four times a day)<br>◯ 4 - 6 hrs<br>◯ 6 - 8 hrs<br>◯ PRN (as needed)<br>◯ Other<br>◯ Unknown
If other, please specify frequency	__________________________
Is medication as needed (PRN)?	◯ Yes<br>◯ No
[cm_med20] Route	◯ PO (by mouth)<br>◯ SC (subcutaneous)<br>◯ IN (intranasal)<br>◯ IM (intramuscular)<br>◯ TOP (topical)<br>◯ Other
If other, please specify route	__________________________
Additional comments about [cm_med20] use	________________________________
