# Supplementary material for: A Survey of Patient-Relevant Outcomes in Pediatric Craniopharyngioma: Focus on Hypothalamic Obesity
Source: Front Endocrinol (Lausanne). 2022 May 9;13:876770. doi: 10.3389/fendo.2022.876770 (PMC9124861; doi:10.3389/fendo.2022.876770)
Supplement: Supplementary file 2 [file Table_1.docx]

Supplementary Material

# Supplementary Tables

**Supplementary Table 1.** Patient demographics and clinical characteristics by each survey component. All 106 caregivers completed the questions on demographics and medical history, 69 completed the detailed medication list, 93 completed the peer relationship questionnaire, and 78 completed the survey on research priorities.

|  | Medical History | Medication List | Peer Relationship | Research Priorities |
| --- | --- | --- | --- | --- |
| Total Patients (%) | n=106 | n=69 | n=93 | n=78 |
| **Age at Diagnosis** | | | | |
| <5 years old  5-11 years old | 23 (22)  70 (66) | 18 (26)  44 (64) | 15 (16)  67 (72) | 19 (24)  49 (63) |
| 12-17 years old  Not Reported | 11 (10)  2 (2) | 7 (10) | 10 (11)  1 (1) | 9 (12)  1 (1) |
| **Current Age** | | | | |
| <5 years old  5-11 years old | 8 (8)  49 (46) | 6 (9)  36 (52) | Excluded  46 (49) | 6 (8)  37 (47) |
| 12-17 years old | 49(46) | 27 (39) | 47 (51) | 35 (45) |
| **Sex** | | | | |
| Male | 65 (61) | 38 (55) | 59 (63) | 46 (59) |
| Female | 41 (39) | 31 (45) | 34 (37) | 32 (41) |
| **Obesity Prior to Craniopharyngioma Diagnosis** | | | | |
| Obese | 6 (6) | 5 (7) | 5 (5) | 5 (6) |
| Non-Obese | 100 (94) | 64 (93) | 88 (95) | 73 (94) |
| **Current Obesity Status** | | | | |
| Obese | 60 (57) | 41 (59) | 55 (59) | 47 (60) |
| Non-Obese | 46 (43) | 28 (41) | 38 (41) | 31 (40) |
| **Treatment** | | | | |
| Endoscopic Surgery | 38 (36) | 26 (38) | 33 (35) | 30 (38) |
| Open Surgery | 71 (67) | 42 (61) | 62 (67) | 48 (62) |
| Cyst Drainage | 25 (24) | 18 (26) | 21 (23) | 20 (26) |
| Radiation | 13 (12) | 8 (12) | 13 (14) | 10 (13) |
| Proton Beam | 35 (33) | 22 (32) | 30 (32) | 24 (31) |
| Chemotherapy | 3 (3) | 2 (3) | 3 (3) | 3 (4) |

**Supplementary Table 2.** Patient demographics and clinical characteristics of survey cohort compared to a CHOP cohort that includes all patients with craniopharyngioma diagnosed between 2008 and 2020 and receiving clinical care at CHOP.

|  | **Survey Cohort**  (n=106) | **CHOP Cohort**  (n=61) |
| --- | --- | --- |
| **Average Age at Diagnosis** | 7.2 years +/- 3.2 (SD) | 8.5 years +/- 3.8 (SD) |
| **Gender** | 61% Male | 61% Male |
| **Radiation** (including proton beam) | 45% Radiation | 38% Radiation |
| **Obesity Pre-Diagnosis** | 5.7% | 28% |
| **Obesity Post-Diagnosis** | 57% | 54% |

**Supplementary Table 3.** Reported pituitary hormone deficiencies of survey cohort compared to subset who completed the medical use questionnaire. The prevalence of pituitary hormone deficiencies is stratified by current obesity status based on caregiver-reported medical history in the survey cohort of 106 caregivers who completed the medical history questions and the subset of these (N=69 of 106) who elected to complete the detailed medication list.

| **Caregiver-reported Hormone Deficiencies (Survey Cohort)** | | | |
| --- | --- | --- | --- |
| **Hormone deficiency** | **Individuals**  **with obesity**  **(n=60)**  **n (%)** | **Individuals without obesity (n=46)**  **n (%)** | **All**  **Individuals**  **(N=106)**  **n (%)** |
| Thyroid | 52 (87) | 41 (89) | 93 (88) |
| Adrenal | 51 (85) | 36 (78) | 87 (82) |
| Growth | 49 (82) | 39 (85) | 88 (83) |
| Gonadal Steroids* | 18 (72) | 13 (81) | 31 (76) |
| Vasopressin | 47 (78) | 37 (80) | 84 (79) |
| Pan-hypopituitarism* | 34 (57) | 26 (57) | 60 (57) |
| **Caregiver-reported Hormone Deficiencies (Medication List Cohort)** | | | |
| **Hormone replacement** | **Individuals with obesity (n=41)**  **n (%)** | **Individuals without obesity (n=28)**  **n (%)** | **All**  **Individuals**  **(N=69)**  **n (%)** |
| Thyroid | 36 (88) | 24 (86) | 60 (87) |
| Adrenal | 34 (83) | 19 (68) | 53 (77) |
| Growth | 34 (83) | 23 (82) | 57 (83) |
| Gonadal Steroids* | 11 (73) | 5 (71) | 16 (73) |
| Vasopressin | 33 (80) | 21 (75) | 54 (78) |
| Pan-hypopituitarism* | 24 (59) | 13 (46) | 37 (54) |

**Supplementary Table 4.** Regression analysis, effects of demographic and clinical factors on peer relationships (PROMIS parent proxy T-score). Linear regression analysis was performed to assess the independent effects of current age, sex, obesity, and radiation on social function (PROMIS parent proxy T-score). β coefficient, along with 95% confidence interval is shown for each input variable. Statistical significance is indicated by *p<0.05; **p<0.001.

| **Variable** | **β (95% CI)** |
| --- | --- |
| Current age (y) | -1.1 (-1.6, -0.5)** |
| Sex  (reference=female) | 3.5 (-0.6, 7.6) |
| Obesity status  (reference=no obesity) | -5.2 (-9.3, -1.2)* |
| Any radiation exposure  (reference=no radiation) | -2.3 (-6.3, 1.8) |

*p < 0.05; **p < 0.001
